# Enrichment and Purification of Deoxyschizandrin and γ-Schizandrin from the Extract of *Schisandra chinensis* Fruit by Macroporous Resins

**DOI:** 10.3390/molecules17033510

**Published:** 2012-03-19

**Authors:** Feng-Jian Yang, Chun-Hui Ma, Lei Yang, Chun-Jian Zhao, Ying Zhang, Yuan-Gang Zu

**Affiliations:** Key Laboratory of Forest Plant Ecology, Northeast Forestry University, Harbin 150040, China; Email: yangfj@nefu.edu.cn (F.-J.Y.); ylmanefu@163.com (C.-H.M.); zcjsj@163.com (C.-J.Z.); cxqnefu@126.com (Y.Z.)

**Keywords:** *Schisandra chinensis*, deoxyschizandrin, γ-schizandrin, macroporous resin, adsorption and desorption, purification

## Abstract

In present study, the performance and separation characteristics of 21 macroporous resins for the enrichment and purification of deoxyschizandrin and γ-schizandrin, the two major lignans from *Schisandra chinensis* extracts, were evaluated. According to our results, HPD5000, which adsorbs by the molecular tiers model, was the best macroporous resin, offering higher adsorption and desorption capacities and higher adsorption speed for deoxyschizandrin and γ-schizandrin than other resins. Columns packed with HPD5000 resin were used to perform dynamic adsorption and desorption tests to optimize the technical parameters of the separation process. The results showed that the best adsorption time is 4 h, the rate of adsorption is 0.85 mL/min (4 BV/h) and the rate of desorption is 0.43 mL/min (2 BV/h). After elution with 90% ethanol, the purity of deoxy-schizandrin increased 12.62-fold from 0.37% to 4.67%, the purity of γ-schizandrin increased 15.8-fold from 0.65% to 10.27%, and the recovery rate was more than 80%.

## 1. Introduction

Fructus Schisandrae Chinensis, the dried fruit of *Schisandra chinensis* (Magnoliaceae) is one of the most famous and frequently used herbal medicines and food additives. It has a long history of medical use in China, Korea and Japan as an astringent, sedative and tonic agent to treat various diseases [1,2,3,4]. It was first recorded in the ancient pharmaceutical book “Shen Nong Ben Tsao Ching” as a superior drug and has been used for thousands of years [5]. It is also used in China in stewing fish and meat and as a flavouring agent and food additive for making soup, tea, yogurt and porridge [6,7,8,9,10]. Various reports have suggested that major bioactive constituents of *S. chinensis* are deoxyschizandrin (DS) and γ-schizandrin (GS) [11,12,13]. Modern pharmacological research has demonstrated that *S. chinensis* shows various beneficial biological active effects, including anti-hepatotoxic, antioxidant, antitumor [14], detoxificant, anticarcinogenic [15], activity on the central nervous system and it counteracts fatigue, and increase endurance [16], has anti-inflammatory properties [17] and improves the physical performance of athletes [16]. The fruit of *S. chinensis* was traditionally used to alleviate or treat diseases due to deficiencies of the lungs, heart and kidneys, imbalance of Yin and Yang, and impairment of Qi [18], such as chronic cough, asthma, spontaneous sweating, palpitation, spermatorrhea, diabetes, insomnia, and forgetfulness. 

The conventional separation method for crude extracts is solid-liquid extraction from natural resources, and then liquid-liquid extraction using different solvents, followed by a column chromatography with a gradient solvent system [19]. However, this method takes a long time, consumes more solvents, and results in lower recovery of the products.

Recently, macroporous resin adsorption technology has been gaining popularity in pharmaceutical applications and has also been used for the purification of herbal crude extracts [20,21,22] because resins have unique adsorption properties and advantages, including ideal pore structure and availability of various surface functional groups, low operation expense, less solvent consumption and easy regeneration [23,24]. For example, macroporous resins have been successfully used in the separation of targeted components from other impurities in crude biological samples. Such as plant polysaccharide [25], morroniside and loganin [26], neohesperidin [27], oleuropein [28] and arctiin [29] and so on.

This study aimed to develop an efficient method for the separation of DS and GS with the optimal resin. The information in this study is significant for the selection of suitable adsorption resins for enrichment and purification of DS and GS from *S. chinensis* or other herbal materials. In general, an efficient separation method with a moderate purification effect can be used economically for recovering and concentrating targeted phytochemicals in industrial practice.

## 2. Results and Discussion

### 2.1. HPLC Chromatograms of S. chinensis Extracts

**Figure 1:**
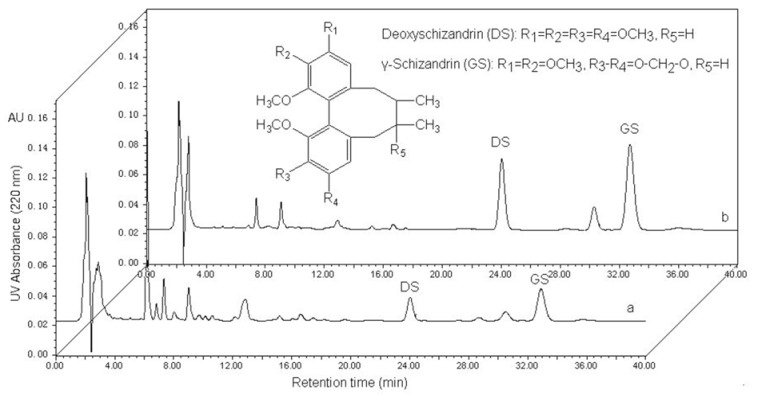
HPLC chromatograms of sample solution of *S. chinensis* extracts (**a**) and desorption solution (**b**) from HPD5000. Inset: The molecular structures of deoxyschizandrin [DS] and γ-schizandrin [GS].

The retention times of DS and GS are 24.9 and 33.8 min, respectively (Figure 1). The corresponding calibration curves for each compound are Y_DS_ = 2.34 × 10^5^X + 4.83 × 10^3^ (r = 0.9999) and Y_GS_ = 3.75 × 10^5^X + 6.11 × 10^3^ (r = 0.9996). A good linearity was found for DS and GS in the range of 0.0108–6.78 and 0.0168–10.5 mg/mL, respectively.

### 2.2. Adsorption and Desorption Capacities of Resins

The selectivity of resins is based on their adsorption and desorption capacities, and the ratio of desorption and the adsorption rates. The following equations were used to quantify the capacities of adsorption and desorption as well as the desorption ratio:

Adsorption evaluation:

*Q_e_*(µg/g) = (*C_0_* − *C_e_*) × *V_i_* (1 − *M*) × *W* (1)

where *Q_e_* is the adsorption capacity at adsorption equilibrium (µg/g anhydrous resin); *C_0_* and *C_e_* are the initial and equilibrium concentrations of DS and GS in the solution, respectively (µg/mL); *V_i_* is the volume of the initial sample solution (mL); *M* is the ratio of water content; *W* is the weight of resin (g).

Desorption evaluation:

*Q_d_* (µg/g) *= C_d_ × V_d_ (1 − M)* × *W* (2)

*D (%) = C_d_ × V_d_ (C_0_ − C_e_)* × *V_i_* × 100% (3)

where *Q_d_* is the desorption capacity after adsorption equilibrium (µg/g anhydrous resin); *C_d_* is the concentration of DS and GS in the desorption solution (µg/mL); *V_d_* is the volume of the desorption solution (mL); *D* is the desorption ratio (%); *C_0_*, *C_e_*, *V_i_* and *M* are the same as described above.

The adsorption capacity and the desorption capacity of all the resins tested in this study are shown in Table 1. In the test, we examined resins with four types of adsorption, including non-polar, weakly polar and polar. 

**Table 1:** Physical properties and absorption characteristics of the test macroporous resins.

Trade name	Polarity	Surface area (m^2^/g)	Average pore diameter (nm)	Particle size(mm)	Specific volume (dm^3^/g)	Moisture contents(%)	*Q_e_* of DS (µg/g)	*Q_e_* of GS (µg/g)	*D* of DS (%)	*D* of GS (%)
HPD80	Non-polar	350–400	80–85	0.3–1.25	1.33-1.54	67.84	499.65 ± 16.45	443.43 ± 15.12	51.64 ± 1.77	84.52 ± 2.77
HPD100	Non-polar	650–700	85–90	0.3–1.25	1.33–1.54	65.00	669.85 ± 22.34	790.26 ± 25.67	48.63 ± 1.66	43.63 ± 1.45
HPD100A	Non-polar	650–700	95–100	0.3–1.25	1.33–1.54	66.67	544.00 ± 19.01	718.96 ± 24.44	50.24 ± 1.67	87.09 ± 2.99
HPD100B	Non-polar	500–580	120–160	0.3–1.25	1.33–1.54	61.49	535.91 ± 18.23	515.43 ± 18.12	74.36 ± 2.55	93.96 ± 3.22
HPD100C	Non-polar	720–760	80–90	0.3–1.25	1.33–1.54	61.68	500.32 ± 16.94	552.55 ± 18.34	70.07 ± 2.39	93.37 ± 3.18
HPD200A	Non-polar	700–750	85–90	0.3–1.25	1.33–1.54	54.90	398.75 ± 13.34	649.62 ± 22.22	75.83 ± 2.61	10.56 ± 0.45
HPD300	Non-polar	800–870	50–55	0.3–1.25	1.33–1.54	75.52	848.13 ± 27.33	809.76 ± 26.76	45.81 ± 1.55	64.96 ± 2.22
HPD700	Non-polar	650–700	85–90	0.3–1.25	1.33–1.54	66.10	561.75 ± 19.11	891.18 ± 30.33	52.51 ± 1.89	71.23 ± 2.45
HPD910	Non-polar	450–550	85–90	0.3–1.25	1.33–1.54	50.00	413.04 ± 14.02	610.84 ± 21.12	61.40 ± 2.11	93.11 ± 3.22
HPD5000	Non-polar	550–600	100–110	0.3–1.25	1.33–1.54	73.28	762.61 ± 22.98	738.68 ± 25.25	93.36 ± 3.18	94.69 ± 3.22
AB-8	Weak-polar	480–520	130–140	0.3–1.25	1.43–1.54	65.00	741.63 ± 26.02	672.28 ± 22.22	44.37 ± 1.55	61.44 ± 2.06
D101	Weak-polar	400–600	100–120	0.3–1.25	1.43–1.54	66.47	702.16 ± 23.88	634.14 ± 21.55	46.03 ± 1.56	76.66 ± 2.75
HPD722	Weak-polar	485–530	130–140	0.3–1.25	1.33–1.54	58.95	641.16 ± 22.92	705.83 ± 23.98	47.21 ± 1.66	82.77 ± 2.88
HPD-D	Polar	650–750	90–110	0.3–1.25	1.33–1.54	73.06	476.34 ± 16.33	568.21 ± 19.45	66.02 ± 2.22	88.07 ± 2.99
HPD200L	Polar	500–550	80–90	0.3–1.25	1.33–1.54	72.86	678.53 ± 23.09	607.93 ± 21.12	50.24 ± 1.73	90.82 ± 3.11
HPD300L	Polar	800–870	50–55	0.3–1.25	1.33–1.54	69.84	578.81 ± 29.43	935.08 ± 31.88	59.39 ± 2.14	84.72 ± 2.86
HPD400	Polar	500–550	75–80	0.3–1.25	1.33–1.54	68.93	535.62 ± 19.33	602.80 ± 20.54	52.22 ± 1.74	89.27 ± 3.06
HPD400A	Polar	500–550	85–90	0.3–1.25	1.33–1.54	72.37	552.43 ± 19.34	474.55 ± 16.19	50.71 ± 1.72	82.35 ± 2.89
HPD450	Polar	500–550	90–110	0.3–1.25	1.33–1.54	72.00	622.94 ± 22.09	684.47 ± 23.23	57.59 ± 1.93	80.89 ± 2.78
HPD450A	Polar	500–550	85–90	0.3–1.25	1.33–1.54	64.06	517.97 ± 18.03	599.50 ± 20.88	55.52 ± 1.87	82.46 ± 2.99
HPD750	Polar	650–700	85–90	0.3–1.25	1.33–1.54	57.58	363.10 ± 13.22	493.03 ± 16.66	71.38 ± 2.44	89.55 ± 3.13

In general, the adsorption capacity is correlated with adsorption type. The DS and GS from *S. chinensis* are both non-polar compounds, so as expected, the non-polar resins HPD5000 and HPD300 had higher adsorption capacity for DS and GS (762.61 ± 22.98 µg/g and 738.68 ± 25.25 µg/g for HPD5000; 848.13 ± 27.33 µg/g and 809.76 ± 26.76 µg/g for HPD300, respectively), but the desorption capacity of HPD300 is weaker than that of HPD5000 (93.36% ± 3.18% and 94.69% ± 3.22% for HPD5000; 45.81% ± 1.55% and 64.96% ± 2.22% for HPD300, respectively). The HPD5000 resin had a bigger average pore diameter than other resins, and this property helped in the desorption process, however, the selectivity of a single solute in a multifold solute system can be decreased due to the increase of average pore diameter, which leads to a high desorption ratio but low adsorption and desorption capacity [30]. In the comprehensive consideration of the adsorption capacity and desorption ratio, HPD5000 resin was suitable to separate DS and GS from *S. chinensis* and consequently, this resin was chosen for further experiments.

### 2.3. Adsorption and Desorption Kinetics on Resins

**Figure 2:**
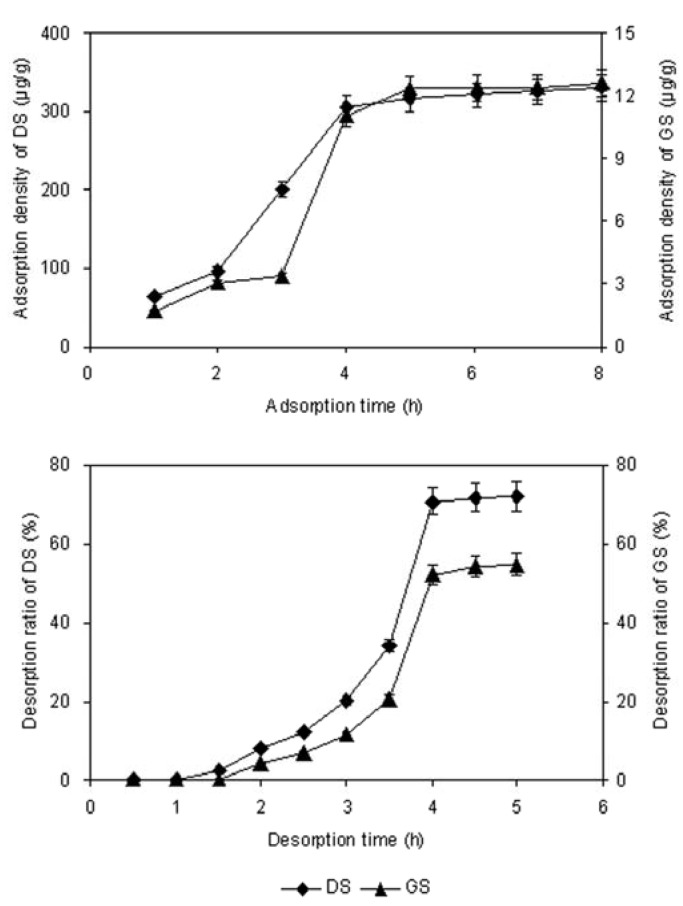
(**a**) The adsorption kinetics curves; (**b**) The desorption kinetics curves.

The adsorption and desorption kinetics curves for DS and GS on HPD5000 resin were obtained. As can be seen from Figure 2a, the adsorption capacity of DS and GS increased with the adsorption time, reaching equilibrium at about 4 h. During the first 3 h, the adsorption capacities increased slowly, and after 3 h increased rapidly, and after 4 h the slopes reached equilibrium. In Figure 2b, the desorption capacity of DS and GS increased with desorption time, reaching equilibrium at about 4 h.

### 2.4. Adsorption Isotherms

**Figure 3:**
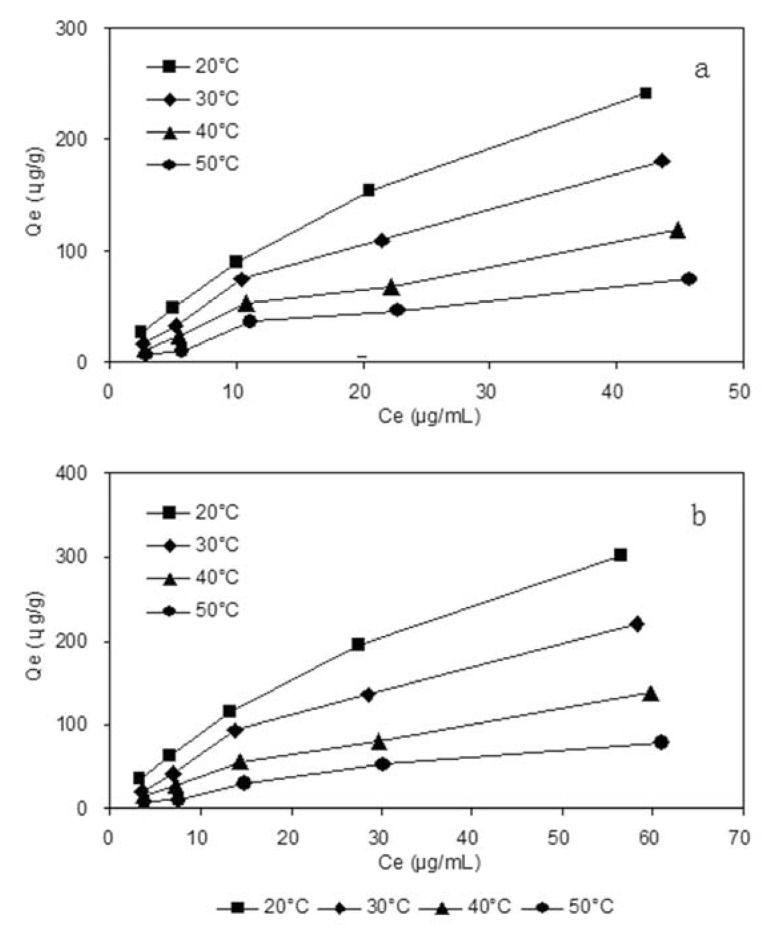
Adsorption isotherms of deoxyschizandrin (**a**) and γ-schizandrin (**b**) on HPD5000 resin at different temperatures.

Adsorption isotherms of DS and GS on HPD5000 resin were investigated at four different temperatures. At the same initial concentration, the adsorption capacities decreased with the temperature increase from 20 to 50 °C, and the adsorption speed was slower than desorption speed, implying that the adsorption process was an exothermic process, and 20 °C was selected as adsorption temperature. We can also see from Figure 3 that the initial concentrations of DS were 2.95, 5.90, 11.81, 23.61 and 47.22 µg/mL, respectively, the initial concentration of GS were 3.91, 7.82, 15.65, 31.30 and 62.60 µg/mL, respectively.

The Langmuir and Freundlich equations are used to reveal the linearity fitting and to describe how solutes interact with the resins. The Langmuir isotherm is the best known and the most frequently used isotherm for the adsorption of solutes from solution. The model of Langmuir can be expressed by the following mathematical formula:

*q_e_ = Q*_m*_C_e_/(K + C_e_) (4)

where *Q_e_* (µg/g) is the concentration of solute per mass of adsorbent (solid phase), also known as adsorptive capacity; *C_e_* (µg/mL) is the concentration of solute in solution (liquid phase) at equilibrium; *K* is the Langmuir constant; *Q_m_* is the empirical constant.

The Freundlich model is an empirical equation, used in the physical adsorption and chemical adsorption for non-ideal adsorption systems. The model of Freundlich can be expressed by the following mathematical formula:

*q_e_ = K C_e_^1/n^* (5)

It can also be expressed by the formula:

*lnQ_e_ = lnk + 1/n lnC_e_* (6)

where *K* is the Freundlich constant that is an indicator of adsorption capacity, and *1*/*n* is an empirical constant related to the magnitude of the adsorption driving force.

The Langmuir and Freundlich parameters summarized in Table 2 indicate the adsorption process was a monomolecular layer adsorption, and the correlation coefficient of the Freundlich equation of DS and GS at 20 °C was 0.9959 and 0.9952, so it could describe the better adsorption behavior of DS and GS on HPD5000 resin.

**Table 2:** Langmuir and Freundlich parameters of DS and GS on HPD5000 resin.

Temperature (°C)	Langmuir equation	R^2^	Freundlich equation	R^2^
DS				
20	qe = 476.19Ce/(42.24 + Ce)	0.9944	qe = 14.22Ce^0.77^	0.9959
30	qe = 434.78Ce/(60.96 + Ce)	0.8803	qe = 8.26Ce^0.84^	0.9800
40	qe = 250.00Ce/(51.63 + Ce)	0.8303	qe = 5.92Ce^0.81^	0.9692
50	qe = 106.38Ce/(23.03 + Ce)	0.9539	qe = 7.11Ce^0.62^	0.9877
GS				
20	qe = 588.24Ce/(53.82 + Ce)	0.9953	qe = 14.86Ce^0.76^	0.9952
30	qe = 500.00Ce/(73.60 + Ce)	0.8849	qe = 8.31Ce^0.83^	0.9770
40	qe = 285.71Ce/(66.77 + Ce)	0.9260	qe = 5.76Ce^0.79^	0.9871
50	qe = 188.68Ce/(83.62 + Ce)	0.9691	qe = 2.77Ce^0.84^	0.9891

### 2.5. Dynamic Leakage Curves on HPD5000 Resin

**Figure 4:**
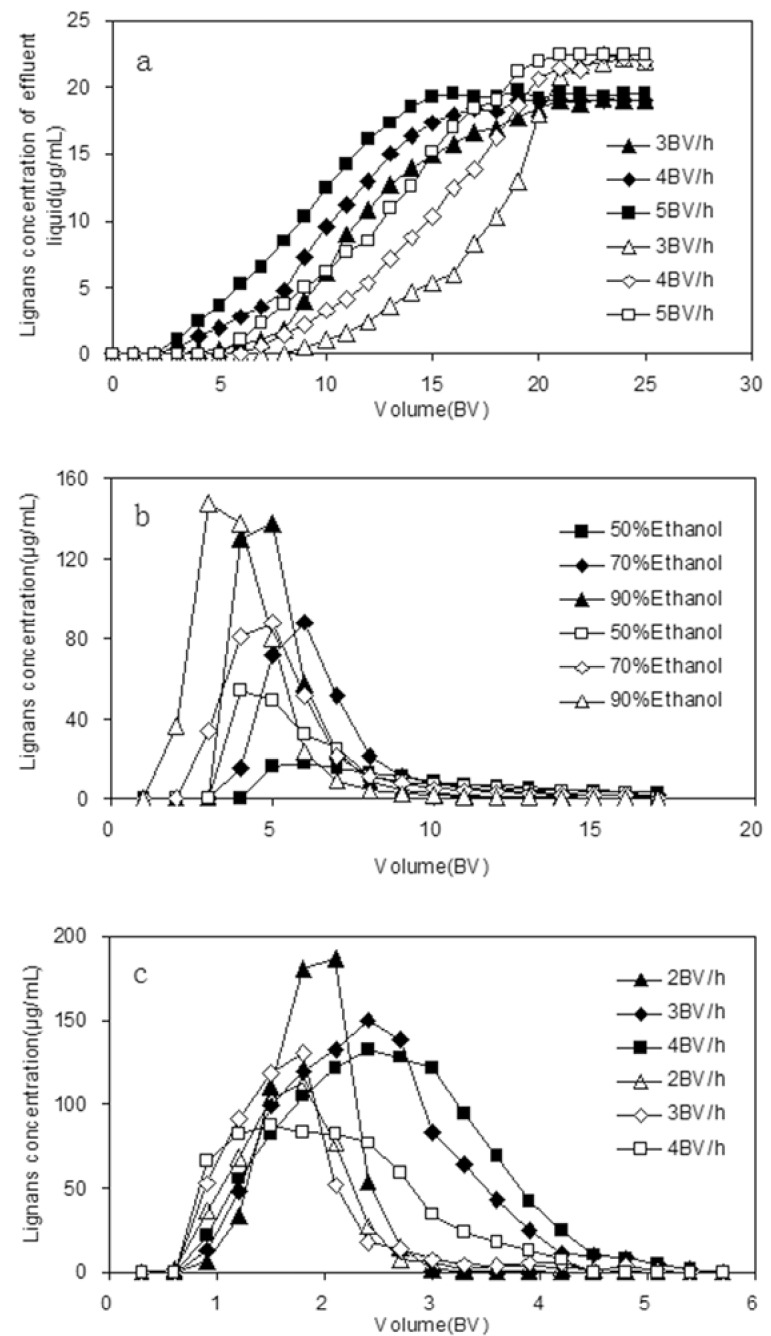
Dynamic leakage curves with different flow rates (**a**), dynamic desorption curves with different ethanol concentrations (**b**) and dynamic desorption curves with different flow rates (**c**).

When the adsorption reaches the break point, the adsorption effect decreases, even disappears, and the solutes leak from the resin, so it is important to set up the leakage curve in order to calculate the quantity of resin needed, the processing volume of sample solution and the proper sample flow rate. The dynamic leakage curves obtained for HPD5000 resin were based on the volume of eluent and the flow rate [31]. The results shown in Figure 4a, indicate that the best adsorption performance was obtained at the lowest flow rate of 3 BV/h, which is likely due to better particle diffusion in sample solutions, but a lower flow rate prolongs the working period, thus, in order to balance the duration of the process and the volume of sample processed, 4 BV/h was chosen as the most appropriate sample flow rate in subsequent experiments. Under this condition the processing volume of sample solution on HPD5000 resin was approximate 13 BV.

### 2.6. Dynamic Desorption Curves on HPD5000 Resin

The dynamic desorption curves using HPD5000 resin were obtained based on the volume of desorption solution and the flow rate of desorption solution. In order to choose a proper desorption solution, different concentrations of ethanol solutions (50%, 70% and 90%) were used to perform desorption tests. In our results, the higher the ethanol concentration, the faster the desorption ratios increased (Figure 4b). Thus for efficiency and economy, 90% ethanol solution was selected as the appropriate desorption solution and used in the dynamic desorption experiments.

The flow rates investigated in this test were 2, 3 and 4 BV/h. As can be seen in Figure 4c, at the flow rate 2 BV/h, DS and GS were totally desorbed in 3 BV; and as the flow rate increased, DS and GS were totally desorbed in 4 and 6 BV at the flow rates of 3 and 4 BV/h, respectively. Consequently, the desorption process of DS and GS finished in 3 BV at the lowest flow rate of 2 BV/h. In conclusion, the lower desorption flow rate made products the more concentrated. Thus, 2 BV/h was selected as the proper desorption flow rate in consideration of the lower volume consumption and high efficiency.

The results revealed the optimum separation process of DS and GS on HPD5000 resin was as follows: (1) adsorption: in sample solution, DS concentration 23.85 µg/mL, GS concentration 31.06 µg/mL, processing volume 13 BV, flow rate 4 BV/h, temperature 20 °C; then washing with deionized water 4 BV, flow rate 4 BV/h, temperature 20 °C; (2) desorption: elution solvent 90% ethanol solution 6 BV and flow rate 2 BV/h. After the separation on HPD5000 resin, the purity of DS increased 12.62-fold from 0.37% to 4.67%, the purity of GS increased 15.8-fold from 0.65% to 10.27%, and the recovery rates were 84.04% for DS and 81.12% for GS.

### 2.7. Adsorption and Desorption Capability of Regenerated HPD5000 Resin

Static adsorption and desorption experiments were performed, in order to study the effect of regeneration times. The adsorption capability and the ratio of desorption of initial resin was defined as 100%. From Table 3, after the resins had been regenerated eight times, the adsorption capability had decreased to 82.44% (DS) and 78.18% (GS) of the initial adsorption capability, and the ratio of desorption had decreased to 83.83% (DS) and 86.27% (GS), thereby indicating that the regenerated resin could still be used for the separation of DS and GS.

**Table 3:** Adsorption and desorption capacity of regenerated HPD5000 resin.

Regenerated HPD5000 Resin	Adsorption capability of DS	The ratio of desorption of DS	Adsorption capability of GS	The ratio of desorption of GS
Initial	100.00%	100.00%	100.00%	100.00%
1 time	97.32%	96.77%	96.33%	96.99%
3 times	94.00%	93.41%	92.87%	92.55%
5 times	88.54%	85.74%	84.70%	88.08%
8 times	82.44%	83.83%	78.18%	86.27%

## 3. Experimental

### 3.1. Chemicals and Reagents

DS and GS standards (98% purity) were purchased from the National Institute for the Control of Pharmaceutical and Biological Products (Beijing, China). The DS and GS standards were dissolved in methanol. Acetonitrile and acetic acid of HPLC grade were purchased from J&K Chemical Ltd. (Beijing, China), and all other solvents and chemicals used in this study were of analytical grade and were obtained from Beijing Chemical Reagents Co. (Beijing, China). Deionized water was purified by a Milli-Q Water Purification system (Millipore, Waltham, MA, USA). All solutions prepared for HPLC were filtered through 0.45 μm membranes before used.

### 3.2. Adsorbents

Tweinty one kinds of macroporous resins were purchased from Cangzhou Bon Adsorber Technology Co., Ltd. (Hebei, China). The physical properties of the macroporous resins are summarized in Table 1. The resins were pretreated by 1 mol/L hydrochloric acid and 1 mol/L sodium hydroxide solutions successively to remove any monomers and porogenic agents trapped inside the pores during the synthesis process. Prior to the adsorption experiments, weighed amounts of resins were soaked in ethanol and subsequently washed thoroughly with deionized water.

### 3.3. Determination of Moisture Content of Resins

Three samples of each kind of macroporous resins were weighed, then placed in a drying oven, and dried at 105 ± 3 °C until the mass did not change. The moisture contents are shown in Table 1.

### 3.4. HPLC Analytical Conditions

The HPLC system consisted of a Waters 717 automatic sample handling system composed of a series HPLC system equipped with 1525 Bin pump, 717 automatic column temperature control box and 2487 UV-detector (Waters, USA). Chromatographic separation for the determination of DS and GS was performed on a HiQ sil-C_18_ reversed-phase column (4.6 mm × 250 mm, 5 μm, KYA TECH). For HPLC analysis, acetonitrile-water-acetic acid (60:40:0.1, v/v/v) is used as the mobile phase with 1.0 mL/min flow rate, 10 μL injection volume, and 20 °C column temperature. The absorbance was measured at a wavelength of 220 nm for the detection of DS and GS, the run time is 45 min.

### 3.5. Preparation of *S. chinensis* Extracts

The fruits of S. chinensis were purchased from San Keshu Trading (Heilongjiang, China). Dried fruits (100 g) were extracted with ethanol-water solution (1,000 mL, 80:20 v/v) at 90 °C for 2 h and filtered. The residue was refluxed with ethanol-water solution (800 mL, 80:20 v/v) for 1.5 h. The ethanol solution of twice extracts (1,000 mL and 800 mL) was filtered and concentrated to dryness under vacuum in a rotary evaporator (RE-52AA, Shanghai Huxi Instrument Co., China), and then dissolved by ethanol-water (50:50, v/v) to get DS and GS solutions of 23.85 and 31.06 µg/mL concentration before using.

### 3.6. Static Adsorption and Desorption Tests

In the static adsorption and desorption experiments, adsorbent (anhydrous resin, 1 g) together with solution (50 mL, 23.85 µg/mL DS and 31.06 µg/mL GS) were added into a flask, and shaken (100 rpm) for 8 h at 20 °C. After adsorption, the resins were washed with deionized water (50 mL) and then static desorption was also performed in the shaker at 20 °C for 5 h. The process was repeated three times. The adsorption isotherm tests on the selected resin were conducted by contacting sample solution (50 mL) at different concentrations with anhydrous resins (1 g), and shaking for 8 h at the temperatures of 20, 30, 40 and 50 °C, respectively.

### 3.7. Dynamic Adsorption and Desorption

Dynamic adsorption and desorption experiments were carried out on glass columns (10 mm × 250 mm) wet-packed with the selected resin (4 g, anhydrous resin basis). All the dynamic experiments were performed at room temperature. The bed volume (BV) of resin was 13 mL. Sample solution flowed through the glass column at the flow rate of 3–5 BV/h. The adsorbate-laden column was washed first with deionized water, and then desorbed with aqueous 90% ethanol solution. Each fraction of desorption solutions was analyzed by HPLC and then concentrated in the rotary evaporation apparatus and dried under vacuum.

### 3.8. Regeneration of HPD5000 Resin

An important property of macroporous resins is their capability of regeneration, which is directly related to the cost of production. The regeneration of HPD5000 was undertaken as follows: first, the exhausted resin samples were washed with 3 BV of ethanol, and then washed successively with 3 BV of 1 mol/L NaOH and 3 BV of 1 mol/L HCl. Finally, the resins were washed with deionized water to a pH value of 6.5–7.0. After the resins had been regenerated eight times, their adsorption densities had decreased to 80.26% of their initial adsorption density, thereby indicating that the regenerated resin could still be used for the separation of DS and GS.

## 4. Conclusions

The separation process of DS and GS with macroporous resin has been successfully developed in this study. Among the 21 resins tested, HPD5000 resin was the most appropriate one for separating DS and GS because of its higher adsorption and desorption capacities, and seen from *R^2^*, their adsorption data fit better to the Freundlich model rather than the Langmuir isotherm. Furthermore, the dynamic adsorption and desorption processes were examined to determine the optimal separation parameters, such as concentration, volume and flow rate of loading sample, adsorption temperature, concentration and volume of eluent, *etc*. The optimum adsorption conditions were as follows: the temperature, the volume, concentration and flow rate of loading sample were 20 °C, 13 bed volumes, 23.85 µg/mL for DS, 31.06 µg/mL for GS, and 4 BV/h, respectively. Desorption was performed with 4 bed volumes of 90% ethanol and the flow rate was 2 BV/h. After concentrating to dryness by rotovaporation after separation on HPD5000 resin, the purity of DS increased 12.62-fold from 0.37% to 4.67%, and the purity of GS increased 15.8-fold from 0.65% to 10.27%, and the recovery rates were 84.04% for DS and 81.12% for GS. Compared to the conventional method, this method offers lower cost, less labor intensiveness, and high separation efficiency. The results will help with the development of plant resources and the application in the food and pharmaceutical industry.
